# Chitosan oligosaccharide enhances the anti-cancer effects of 5-fluorouracil on SNU-C5 colorectal cancer cells by activating ERK

**DOI:** 10.32604/or.2024.052003

**Published:** 2025-03-19

**Authors:** JI-SU HAN, HYE-JIN BOO, JIN WON HYUN, HEESANG SONG, IN-YOUB CHANG, SANG-PIL YOON

**Affiliations:** 1 Jeju Research Center for Natural Medicine, Jeju National University, Jeju, 63243, Republic of Korea; 2 Department of Biochemistry and Molecular Biology, Chosun University School of Medicine, Gwangju, 61452, Republic of Korea; 3 Department of Anatomy, Chosun University School of Medicine, Gwangju, 61452, Republic of Korea; 4 Department of Anatomy, College of Medicine, Jeju National University, Jeju, 63243, Republic of Korea

**Keywords:** Chitosan oligosaccharide (COS), Colorectal cancer (CRC), 5-Fluorouracil (5-FU), ERK (Extracellular signal-regulated protein kinase)

## Abstract

**Background:**

Chitosan oligosaccharide (COS) is the major degradation product of chitosan by enzymatic processes. COS, with complete water solubility, exerts significant biological effects, including anti-cancer activity. We investigated the anti-tumor effects of COS on colorectal cancer as effective therapeutic methods with low side effects are lacking.

**Methods:**

COS was obtained from low molecular weight chitosan by an enzymatic method and the anti-cancer effects were measured by cell viability assay, flow cytometry analysis, Western blotting, and xenograft.

**Results:**

COS suppressed the proliferation of SNU-C5 cells compared to other colorectal cancer cells, but higher concentrations were required in the xenograft model. Co-treatment with 5-fluorouracil (5-FU) and COS enhanced the anti-cancer effects of 5-FU in SNU-C5 cells *in vitro* and *in vivo*. Flow cytometry revealed that COS induced cell cycle arrest at the G0/G1 phase without 5-FU or at the S and G2/M phases with 5-FU but did not affect cell death pathways. COS increased extracellular signal-regulated protein kinase (ERK) activation with or without 5-FU, whereas 5-FU treatment increased p53 activation. A low-dose of an ERK inhibitor suppressed COS-induced ERK activation and resulted in higher proliferation compared with COS.

**Conclusions:**

These results suggest that COS might enhance the anti-cancer effects of 5-FU in SNU-C5 colorectal cancer cells by activating ERK.

## Introduction

Colorectal cancer (CRC) is the 2nd leading cause of cancer development (11.8%) and the 3rd most common cause of cancer death (11.0%) in the Republic of Korea. Although the CRC diagnosis rate (56.7 ± 2.9/100,000 people) has not changed considerably, the crude mortality rate (16.4 to 17.9/100,000 people) has increased over the last decade [1]. The Korean Statistical Information Service considers these changes to be due to the aging of the population. However, related research is needed to overcome the current situation.

The efficacy of cancer chemotherapy is limited by drug resistance to 5-fluorouracil (5-FU)-based chemo-therapeutic agents [2,3]. A previous report [4] summarized the mechanisms of resistance to 5-FU as including reduced drug influx and increased drug efflux, protective autophagy, increased epithelial-mesenchymal transition, cancer stem cells, increased reactive oxygen species, dysregulations of microRNA, epigenetic alterations, increased DNA damage repair, and the tumor microenvironment.

The precise molecular mechanisms of drug resistance in CRC are unknown. Irregularities in protein kinase B (Akt) and extracellular signal-regulated protein kinase (ERK) have been suggested in the development of multidrug resistance [5]. This study focused on 5-FU to identify the possible mechanisms to overcome in drug-resistance in CRC cells. Yeast extract inhibited the proliferation of CRCs and induced poly (ADP-ribose) polymerase-dependent apoptosis by activating the p38-p53-p21 cascade, whether or not resistance to 5-FU was acquired [6]. *Orostachys japonica* showed anti-proliferative effects in 5-FU-resistance acquired SNU-C5 (SNU-C5/5-FUR) cells, but not in wild-type cells, by activating mitogen activated protein kinase (MAPK) signaling pathways, especially ERK and p38 [7].

Chitosan oligosaccharide (COS) has water-soluble properties compared to chitin and chitosan and exerts various biological activities [8,9]. Naveed et al. [10] summarized the mechanisms of the anti-tumor effects of COS. COS induced the production of antioxidant enzymes and AMP-activated protein kinase, which resulted in cell cycle arrest. In CRC, the anti-tumor effects of COS were first reported in HT29 cells, in which inflammatory responses were inhibited [11,12]. Thereafter, COS was reported to promote radiosensitivity with increased apoptosis in SW480 cells [13,14]. COS induced apoptosis and promoted mitosis with S phase arrest in HCT116 cells [15] and in xenograft models [16].

Despite exerting various biological activities in treating tumors, the precise mechanism of the anti-cancer effects of COS on CRC remains not well understood. We focused on a previous report [8] where combined treatment of COS and gemcitabine produced synergistic immune reactions in CT26-bearing tumors. COS induced T cell infiltration in tumors, and combined treatment with gemcitabine prevented the expression of programmed cell death-ligand 1 (PD-L1) compared to gemcitabine treatment. Thus, combining COS with gemcitabine led to better tumor remission by suppressing PD-L1 expression and enhancing immune reactions.

Therefore, the aim of this study was to investigate whether COS exerted anti-cancer effects on CRCs, and whether COS might enhance the anti-cancer effects of 5-FU. Accordingly, the possible underlying mechanisms were examined in MAPK signaling pathways in SNU-C5 cells based on our previous reports [7,17].

## Materials and Methods

### Preparation of COS

Water-soluble COS was obtained by an enzymatic method [9] from low molecular weight chitosan (YB Bio; Gyungbuk, Republic of Korea). The lyophilized COS was dissolved in distilled water immediately before use.

### Reagents and antibodies

MTT (#M6494) was purchased from ThermoFisher Scientific (Seoul, Republic of Korea). 5-FU (#F6627) was obtained from Sigma-Aldrich (Merck KGaA, Darmstadt, Germany). PD98059 (#9900), an inhibitor of ERK, was purchased from Cell Signaling Technology Inc. (Beverly, MA, USA).

c-Jun N-terminal kinase (JNK; #9252), phosphor-Akt (#3787), phosphor-ERK (#4370), phosphor-JNK (#9251), p38 (#9212), and phosphor-p38 (#9211) antibodies were purchased from Cell Signaling Technology Inc.; Akt (#sc-8312), ERK (#sc-93), GAPDH (#sc-47724), and phosphor-p53 (#sc-135630) antibodies were obtained from Santa Cruz Biotechnology (Santa Cruz, CA, USA); and p53 (#GTX70218) antibody was purchased from GeneTex (Irvine, CA, USA).

### Cell culture

HCT116, HT29, and SNU-C5 cell lines (Korean Cell Line Bank; Seoul, Republic of Korea) and the SNU-C5/5-FUR cell line (Research Center for Resistant Cells; Chosun University, Gwangju, Republic of Korea) were cultured according to the supplier’s protocol at 5% CO_2_, 37°C, and humidified atmosphere conditions. The mycoplasma test was done when KCLB distributed the cell lines.

### Cell viability assay

Cells were seeded in triplicate wells of 96-well plates, and treated with COS (0, 1, 10, 100, and 100 μg/mL), 5-FU (0, 0.1, 1, 10, and 100 μM), and PD98059 (0, 1, 5, 10, 25, and 50 μM). Cell density were 2 × 10^3^ cells for HCT116, HT29, and SNU-C5 cells and 5 × 10^3^ cells for SNU-C5/5-FUR cells for the cell viability assay. The cells were incubated for 24 h or 3 days, and MTT solution was added to each well. After 3 h, formazan crystals were dissolved in dimethylsulfoxide (DMSO). The absorbance was read at 595 and 620 nm using a VERSAmax microplate reader (Molecular Devices Korea LLC; Seoul, Republic of Korea). The absorbance values of treated cells were compared to those of vehicle-treated cells, representing 100% cell viability.

### Subcutaneous tumor xenograft model

7-week-old male athymic BALB/c mice were purchased from OrientBio (Seongnam, Republic of Korea) and housed in the Laboratory Animal Facilities, Jeju National University. The mice were housed in a specific pathogen-free environment at a standard temperature (24 ± 2°C), with controlled humidity (50 ± 5%) and 12-h light/dark cycles, and provided standard rodent food and water. All animal experiments were approved by the Jeju National University Institutional Animal Care and Use Committee (IACUC 2019-0051 and IACUC 2022-04). Tumor xenografts were generated by injecting 1 × 10^6^ SNU-C5 cells in 100 μL of Matrigel (BD Biosciences; Seoul, Republic of Korea) into the right flank of the mice as previously described [7].

**Figure 1:**
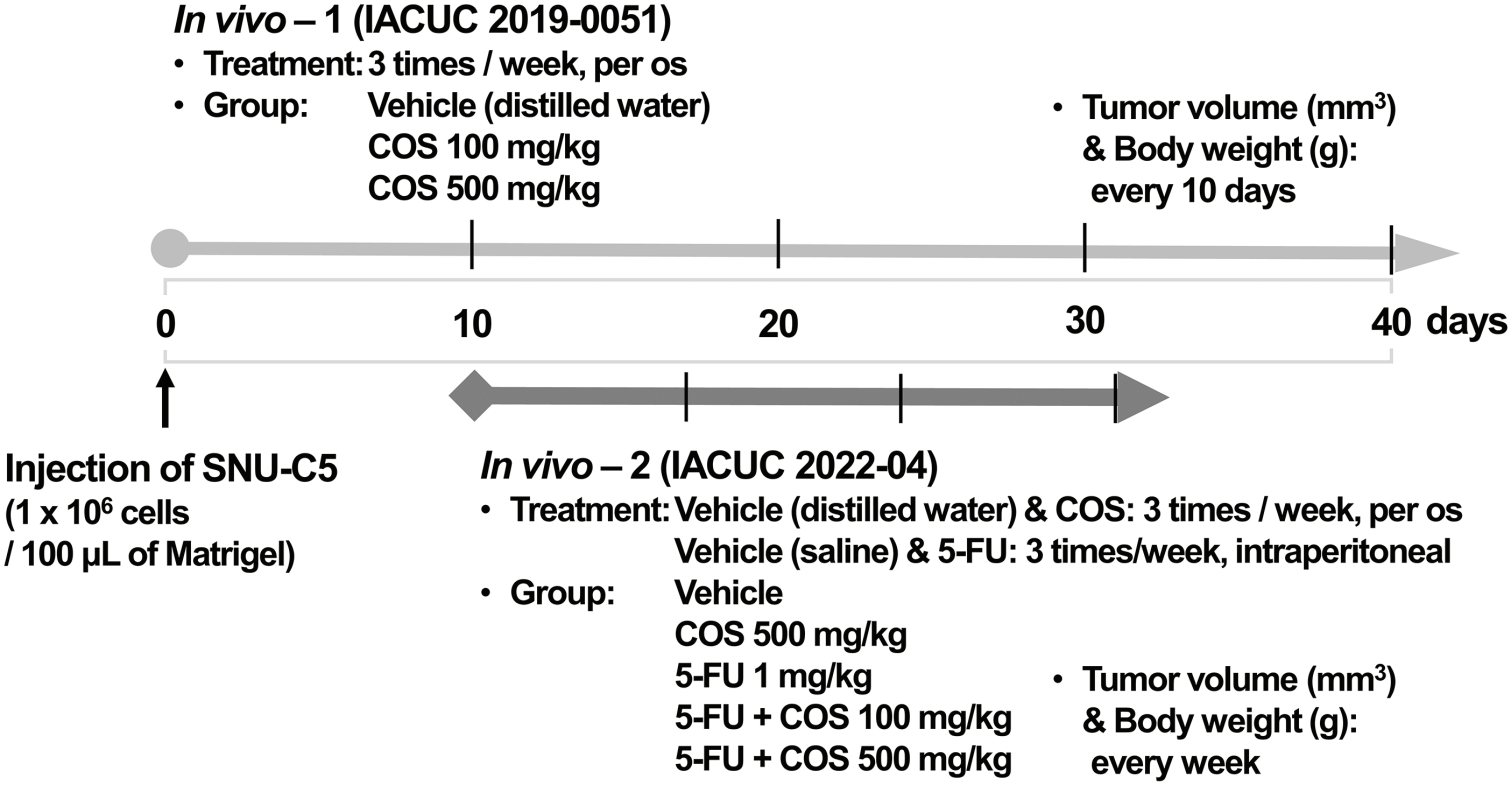
Scheme of xenograft model.

At first (IACUC 2019-0051), the animals were randomly divided into 3 groups (n = 9/group) from the day of injection as follows: control mice treated with distilled water as the vehicle, and COS (100 and 500 mg/kg)-treated mice. Next (IACUC 2022-04), the tumor-bearing mice were randomly divided into 5 groups (n = 9/group) 10 days after the injection, as follows: control group treated with vehicle, COS5 (500 mg/kg), 5-FU (1 mg/kg), 5-FU + COS1 (100 mg/kg), and 5-FU + COS5. Treatment and measurements are described in Fig. 1. Resected tumor weights (g) were measured in euthanized mice.

### Flow cytometry

Cells were treated vehicle, COS (100 μg/mL) and 5-FU (1 μM) for 72 h, and analyzed by the FACSCalibur^TM^ system (BD Biosciences) as described previously [6].

### Western blot analysis

Cells were treated with vehicle, COS (100 μg/mL), 5-FU (1 μM), or PD98059 (10 μM), followed by Western blotting as described previously [6,7].

Cell lysates were subjected to gel electrophoresis with a polyvinylidene difluoride membrane. The membranes were incubated with primary antibodies and the appropriate secondary antibody (#PI-2000 and #PI-1000; Vector Laboratories Inc., Burlingame, CA, USA). Protein bands were detected using the Azure^TM^ c300 and quantified using the AzureSpot analysis software (version 14.2; Azure Biosystemc Inc., Dublin, CA, USA).

### Statistical analysis

All data were compiled from a minimum of 3 replicate experiments and are expressed as the mean ± SEM. A *p*-value of < 0.05, as determined using the Student’s *t*-test or one-way analysis of variance followed by a *post-hoc* test (MS Excel, 2016), indicates a statistically significant difference.

## Results

### Anti-cancer effects of COS in colorectal cancer cells

**Figure 2:**
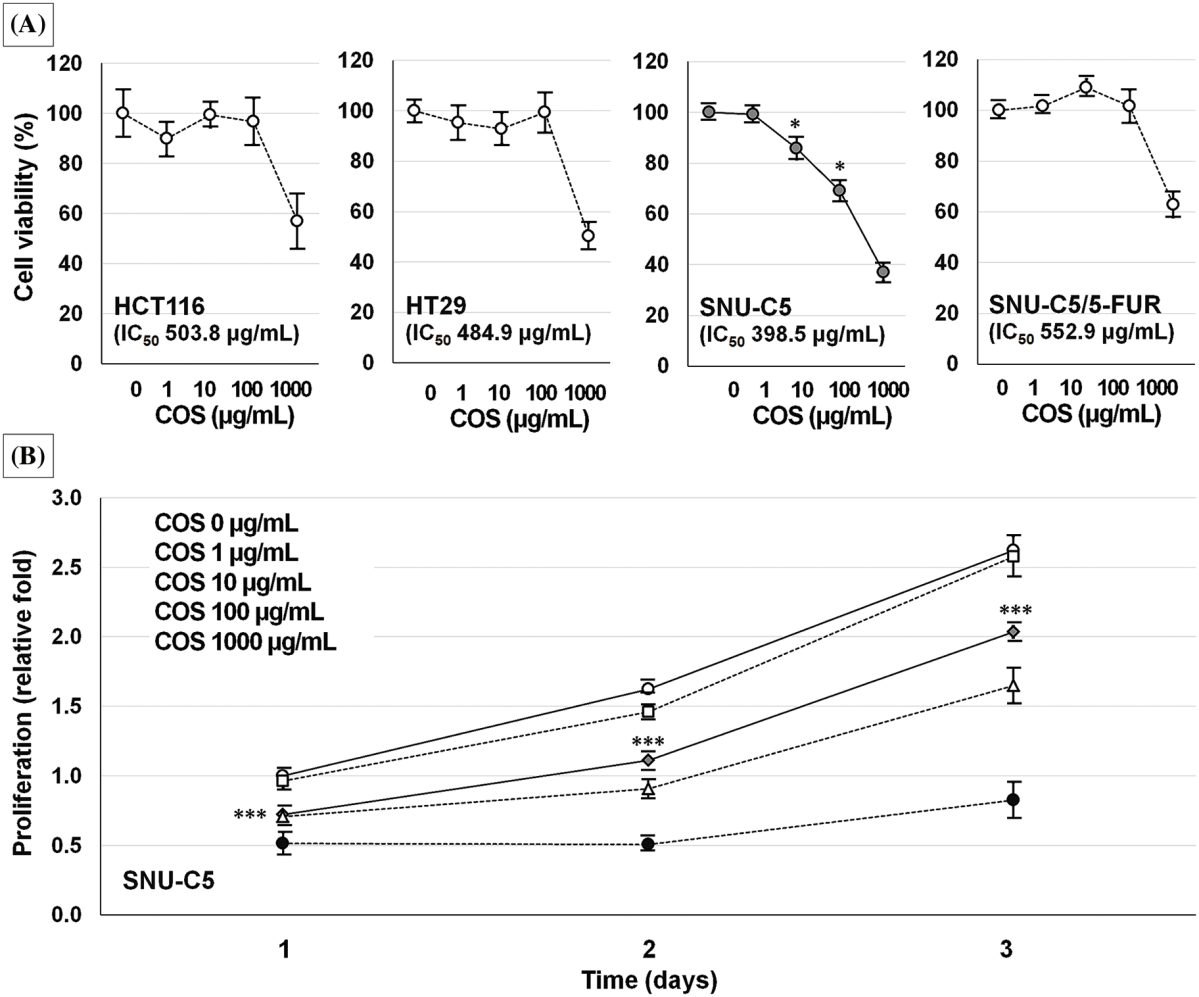
Anti-cancer effects of chitosan oligosaccharide (COS) in colorectal cancer cells. Among the colorectal cancer cell lines tested, SNU-C5 cells were the most sensitive to COS treatment in a dose-dependent manner (A). The anti-proliferative effects were significant at 10 μg/mL of COS (B). *p < 0.05, ***p < 0.001 *vs*. vehicle.

The IC_50_ values of SNU-C5 cells were significantly lower than those of other CRC cells (Fig. 2A). Cell viability of SNU-C5 cells significantly reduced at 10 (85.8% ± 4.3%, *p* = 0.020) and 100 μg/mL (69.1% ± 4.0%, *p* = 0.014) COS, respectively. SNU-C5 cells proliferated up to 1.62 ± 0.01 and 2.62 ± 0.04-fold at 48 and 72 h as compared with the vehicle-treated condition after 24 h of incubation (Fig. 2B). Exposure to 10 μg/mL COS, the minimum effective dose, inhibited cell proliferation by 0.72 ± 0.01, 1.11 ± 0.01, and 2.03 ± 0.04-fold at 24, 48, and 72 h compared to vehicle-treated condition (*p* < 0.001).

**Figure 3:**
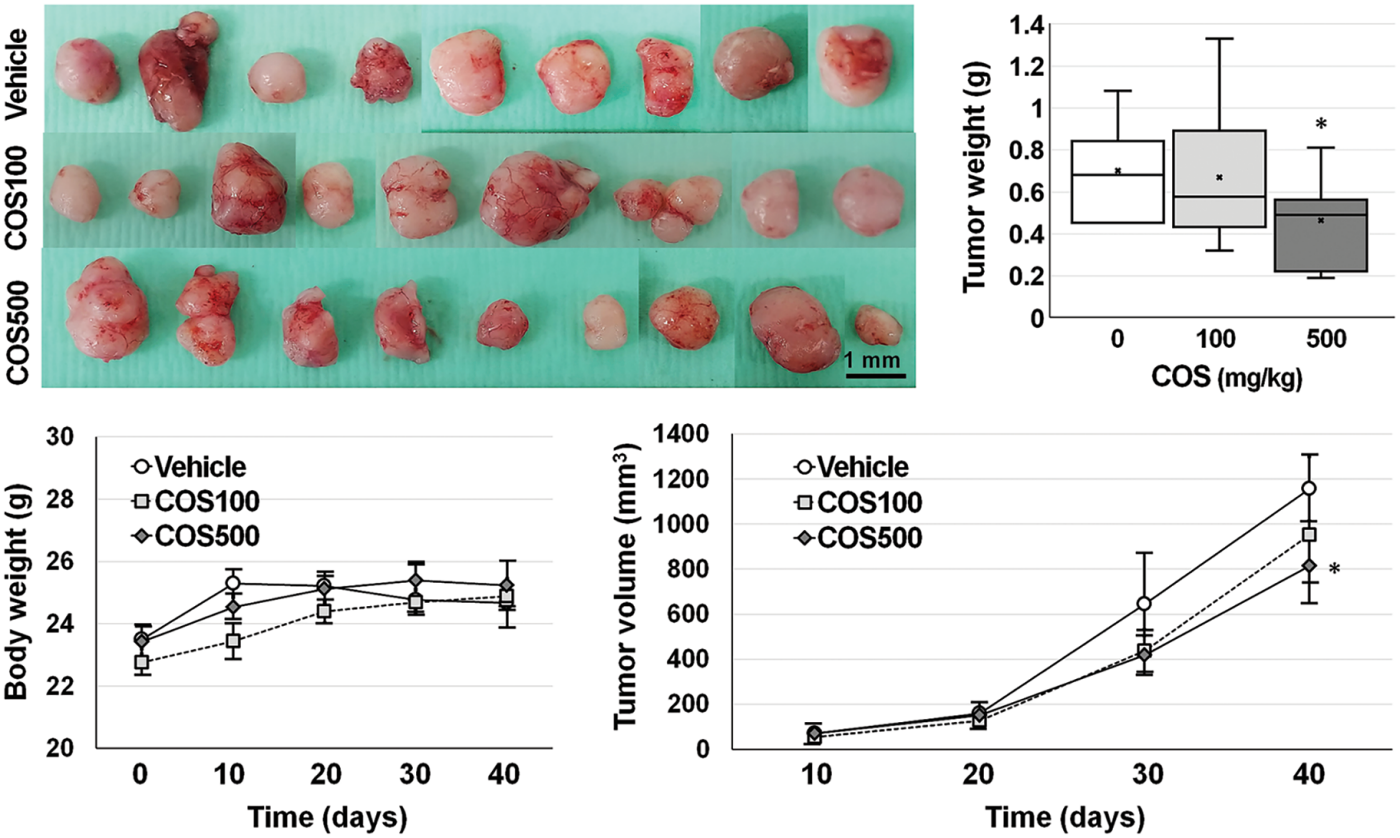
Anti-cancer effects of chitosan oligosaccharide (COS) in SNU-C5 cells *in vivo*. Body weights of SNU-C5 xenograft mice remained stable after COS treatment. COS treatment (500 mg/kg) significantly inhibited tumor growth (volume and weight) 40 days after treatment. *p < 0.05 *vs*. vehicle.

A COS dose of 100 and 500 mg/kg was adopted for the *in vivo* experiment (Fig. 3). Body weights of the mice were similar among the groups throughout the experiment. Each group showed similar tumor volumes at the beginning, which were not different among the groups until day 30. On day 40, the tumor volume in the vehicle, COS 100 mg/kg, and COS 500 mg/kg group were 1156.4 ± 141.9, 1161.0 ± 251.2, and 686.0 ± 116.3 mm^3^ (*p* = 0.144). However, COS treatment at 500 mg/kg was considerably reduced in tumor volume compared with vehicle-treated mice (*p* = 0.012). Similar results were also observed in tumor weights (0.70 ± 0.08, 0.67 ± 0.15, and 0.46 ± 0.08 g), in which COS at 500 mg/kg significantly reduced tumor weights compared with the vehicle-treated group (*p* = 0.010). Taken together, the anti-proliferative effects of COS were observed at higher doses.

### Anti-cancer effects of COS as a 5-FU adjuvant in colorectal cancer cells

**Figure 4:**
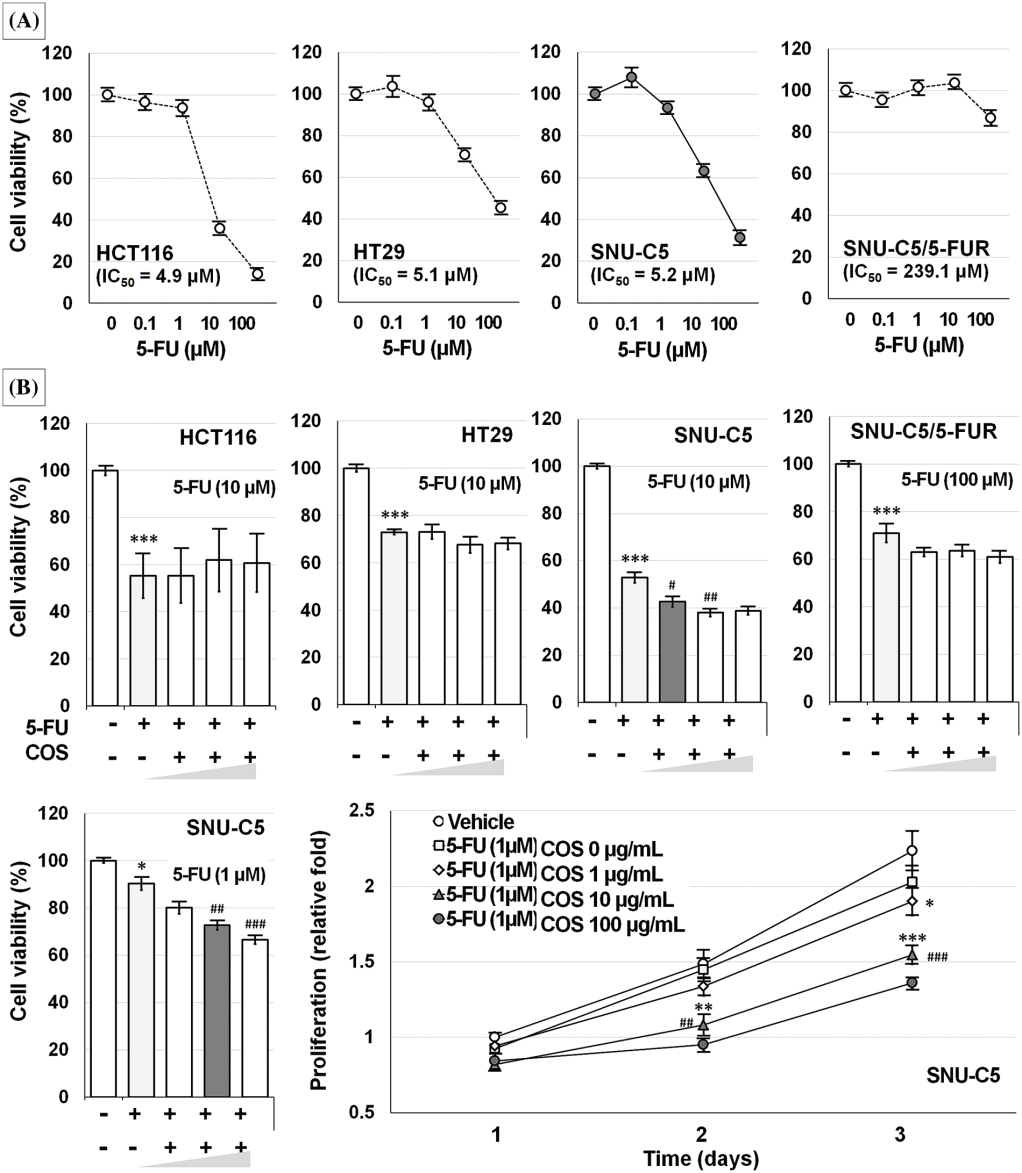
Anti-cancer effects of 5-fluorouracil (5-FU) in colorectal cancer cells. Among the colorectal cancer cell lines tested, SNU-C5/5-FUR cells were the most resistant to 5-FU treatment (A). Co-treatment with 5-FU and chitosan oligosaccharide (COS; 1, 10, and 100 μg/mL) further decreased SNU-C5 cell viability (B). The anti-proliferative effects of co-treatment were significant at 10 μg/mL of COS. *p < 0.05, **p < 0.01, ***p < 0.001 *vs*. vehicle; ^#^*p* < 0.05, ^##^*p* < 0.01, ^###^*p* < 0.001 *vs*. 5-FU.

The IC_50_ value of SNU-C5/5-FUR cells treated with 5-FU was significantly higher than that of other 5-FU-treated CRC cells (Fig. 4A). SNU-C5 cells treated with 1 μM (94.9% ± 1.0%, *p* = 0.001) 5-FU showed significantly lower cell viability whereas other CRC cells did not show considerable changes at this concentration. Co-treatment with 5-FU and COS did not induce any further effects in CRC cells, but showed significant additive effects in SNU-C5 cells (*p* < 0.001) (Fig. 4B). The combined effects of 5-FU and COS were evaluated in SNU-C5 cells using 1 μM 5-FU as the minimally effective dose. SNU-C5 cells proliferated up to 1.49 ± 0.19 and 2.23 ± 0.13-fold at 48 and 72 h compared with vehicle-treated cells following a 24 h incubation. 5-FU treatment inhibited proliferation by 1.45 ± 0.08 and 2.03 ± 0.11-fold at 48 and 72 h compared with vehicle-treated cells. Co-treatment with 5-FU and 10 or 100 μg/mL COS inhibited proliferation by 1.08 ± 0.07 (*p* = 0.001) and 0.95 ± 0.05-fold (*p* < 0.001) at 48 h, and 1.55 ± 0.06 and 1.36 ± 0.03-fold (*p* < 0.001/each) at 72 h, respectively.

**Figure 5:**
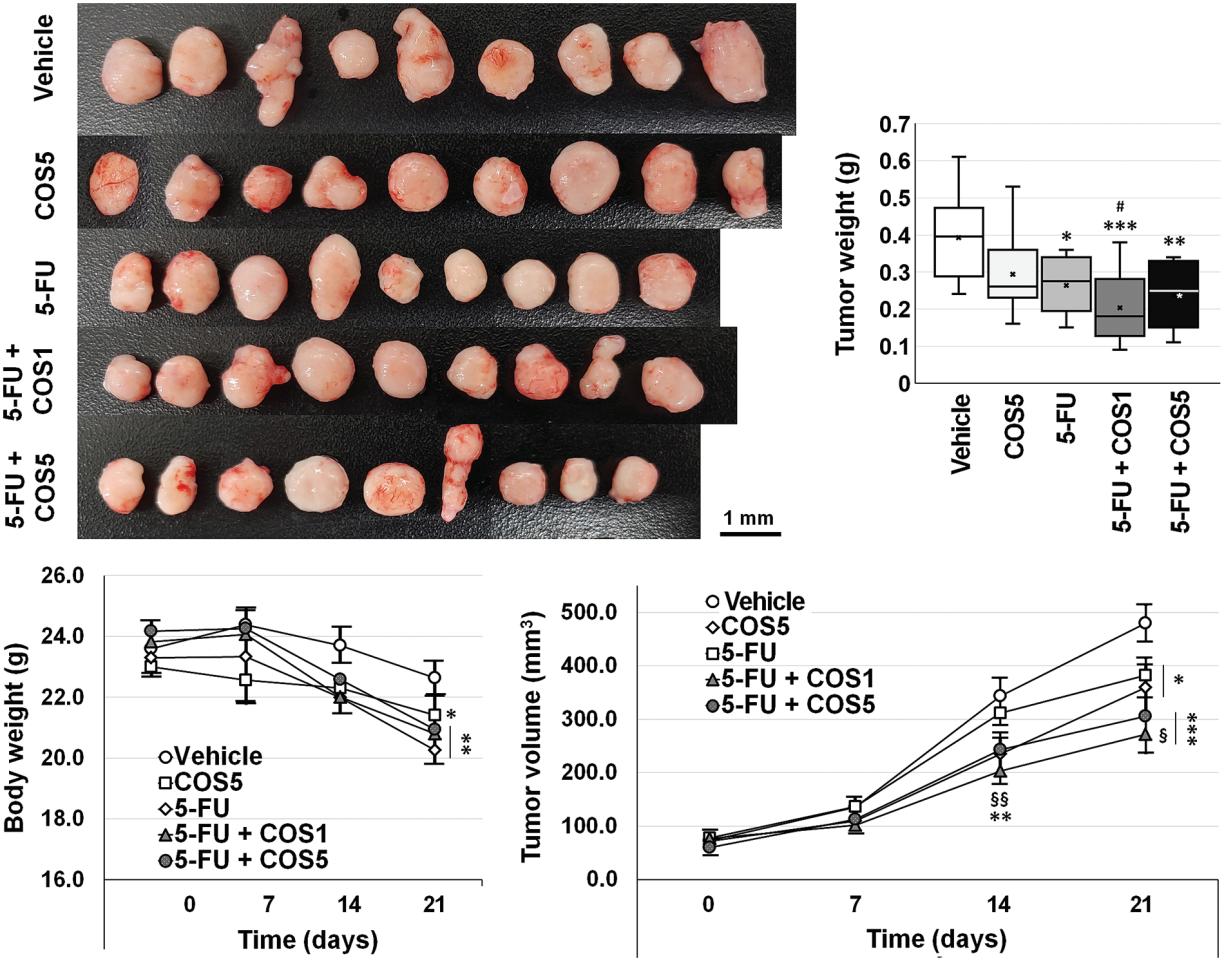
Anti-cancer effects of chitosan oligosaccharide (COS) as an adjuvant to 5-fluorouracil (5-FU) in SNU-C5 cells *in vivo*. Co-treatment with 5-FU and chitosan oligosaccharide (COS) further decreased SNU-C5 cell viability. The anti-proliferative effects of co-treatment were significant at 100 μg/mL of COS. *p < 0.05, **p < 0.01, ***p < 0.001 *vs*. vehicle; ^#^*p* < 0.05 *vs*. COS; ^§^*p* < 0.05, ^§§^*p* < 0.01 *vs*. 5-FU.

COS at 500 mg/kg was adopted for the *in vivo* experiment (Fig. 5). Body weights of the mice in 5-FU-injected groups were decreased, and thus, the experiments were terminated 3 weeks after treatment.

Each group showed similar tumor volumes at the beginning. Single and co-treatment with 5-FU and COS significantly reduced tumor volumes on day 21 (*p* = 0.002). COS5 (359.3 ± 41.6 mm^3^, *p* = 0.019) and 5-FU (381.8 ± 32.3 mm^3^, *p* = 0.026) treatment reduced tumor volumes compared with vehicle-treated mice (479.5 ± 34.3 mm^3^). Co-treatment of COS1 (271.6 ± 35.1 mm^3^, *p* < 0.001) or COS5 (305.2 ± 33.4 mm^3^, *p* < 0.001) with 5-FU showed further significant reductions in tumor volumes. Co-treatment with 5-FU and COS1 showed considerable changes (*p* = 0.017) compared to the 5-FU group, but the changes were not significantly different from the COS5 group (*p* = 0.062). Co-treatment with 5-FU and COS5 did not show significant changes compared to the COS5 or 5-FU groups. Similar results were also observed for tumor weights (0.39 ± 0.04, 0.29 ± 0.04, 0.26 ± 0.02, 0.20 ± 0.03, and 0.24 ± 0.03 g, respectively, for vehicle, COS5, 5-FU, 5-FU + COS1, and 5-FU + COS5 group). All treated groups showed considerable reductions in tumor weight (*p* = 0.039, *p* = 0.004, *p* = 0.001, *p* = 0.001 as previously described in each group *vs*. vehicle-treated group).

### Anti-cancer effects of COS through cell cycle arrest

**Figure 6:**
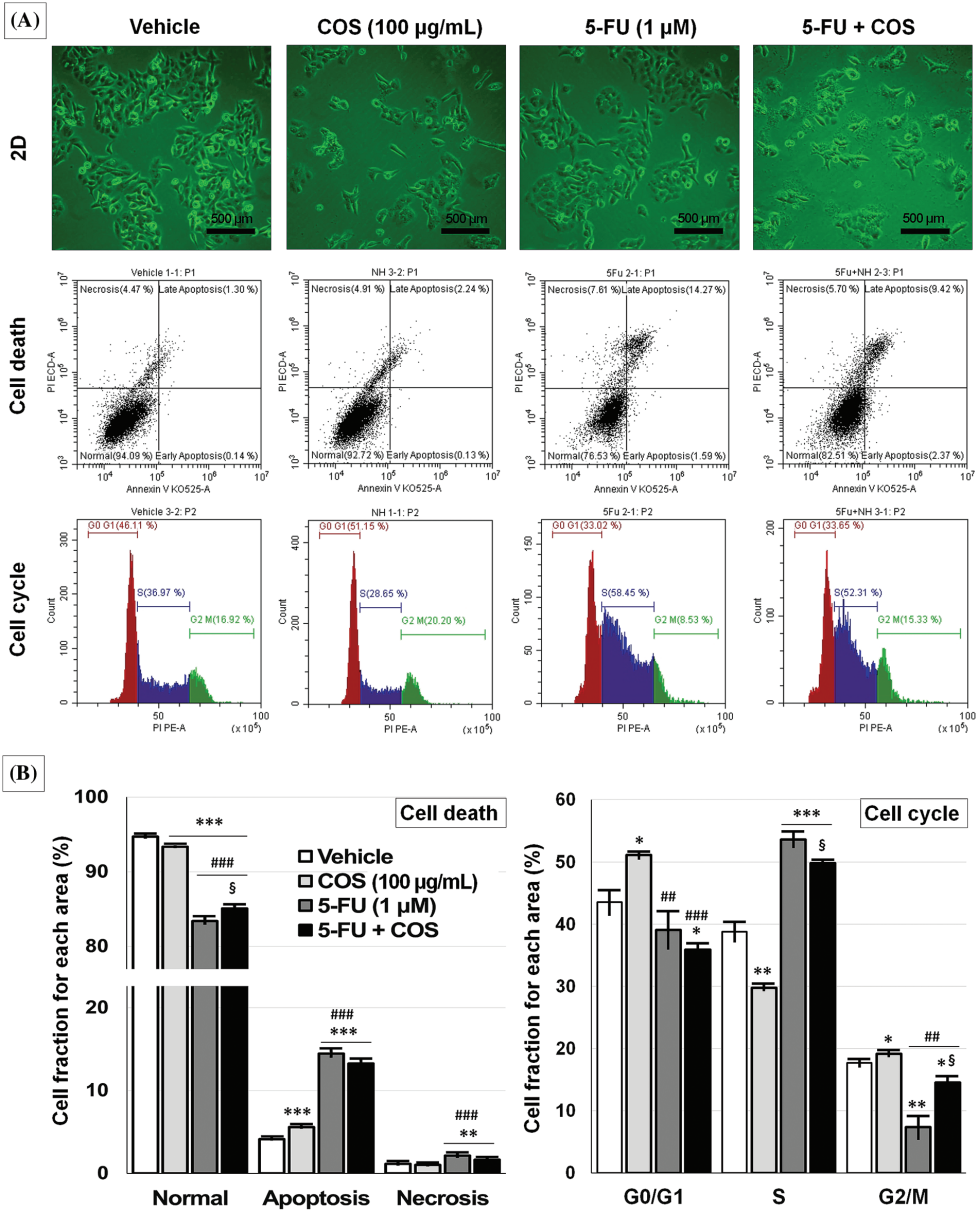
Anti-cancer effects of chitosan oligosaccharide (COS) on cell cycle arrest in SNU-C5 cells. Representative images of the vehicle, COS (100 μg/mL), 5-FU (1 μM), and 5-FU + COS-treated SNU-C5 cells and flow cytometry (A). COS did not affect cell death pathways, whereas 5-FU induced apoptosis regardless of COS co-treatment. COS induced cell cycle arrest at the G0/G1 phase without 5-FU and the S and G2/M phases with 5-FU (B). *p < 0.05, **p < 0.01, ***p < 0.001 *vs*. vehicle; ^##^*p* < 0.01, ^###^*p* < 0.001 *vs*. COS; ^§^*p* < 0.05 *vs*. 5-FU.

As COS enhanced the anti-cancer effects of 5-FU in SNU-C5 cells, the cells were incubated with vehicle, COS (100 μg/mL), 5-FU (1 μM), and COS plus 5-FU, then analyzed by flow cytometry (Fig. 6). COS treatment resulted in a slight change in cell death compared with vehicle- or 5-FU-treated cells. 5-FU treatment markedly increased the ratio of apoptosis and necrosis in SNU-C5 cells with or without COS. Increased cell fraction was observed in total apoptosis (4.14 ± 0.04, 5.60 ± 0.08, 14.46 ± 0.35, and 13.26% ± 0.47% for each treatment described above; *p* < 0.001), and necrosis (1.16 ± 0.06, 1.04 ± 0.06, 2.15 ± 0.12, and 1.65% ± 0.09% for each treatment described above; *p* < 0.001) after 5-FU treatment.

Cell cycle analysis revealed a significant change in the G0/G1 and G2/M phases in COS-treated SNU-C5 cells. 5-FU treatment considerably increased the fraction of cells in the S phase, and the fraction was decreased with COS treatment regardless of 5-FU treatment (38.76 ± 1.49, 29.76 ± 0.31, 53.58 ± 1.19, and 49.82% ± 0.19% for each treatment described above; *p* < 0.001). COS treatment without 5-FU significantly increased the fraction of cells in the G0/G1 phase (*p* = 0.010), but this was slightly decreased with 5-FU (*p* = 0.184) (43.53 ± 1.96, 51.08 ± 0.52, 39.08 ± 3.01, and 35.87% ± 0.95% for each treatment described above; *p* = 0.002). COS treatment without 5-FU slightly increased the fraction of cells in the G2/M phase (17.71 ± 0.54 *vs*. 19.16% ± 0.36%; *p* = 0.044), but 5-FU co-treatment showed a marked increase (7.34 ± 1.83 *vs*. 14.53% ± 0.99%; *p* = 0.013).

### Anti-cancer effects of COS by activating ERK

**Figure 7:**
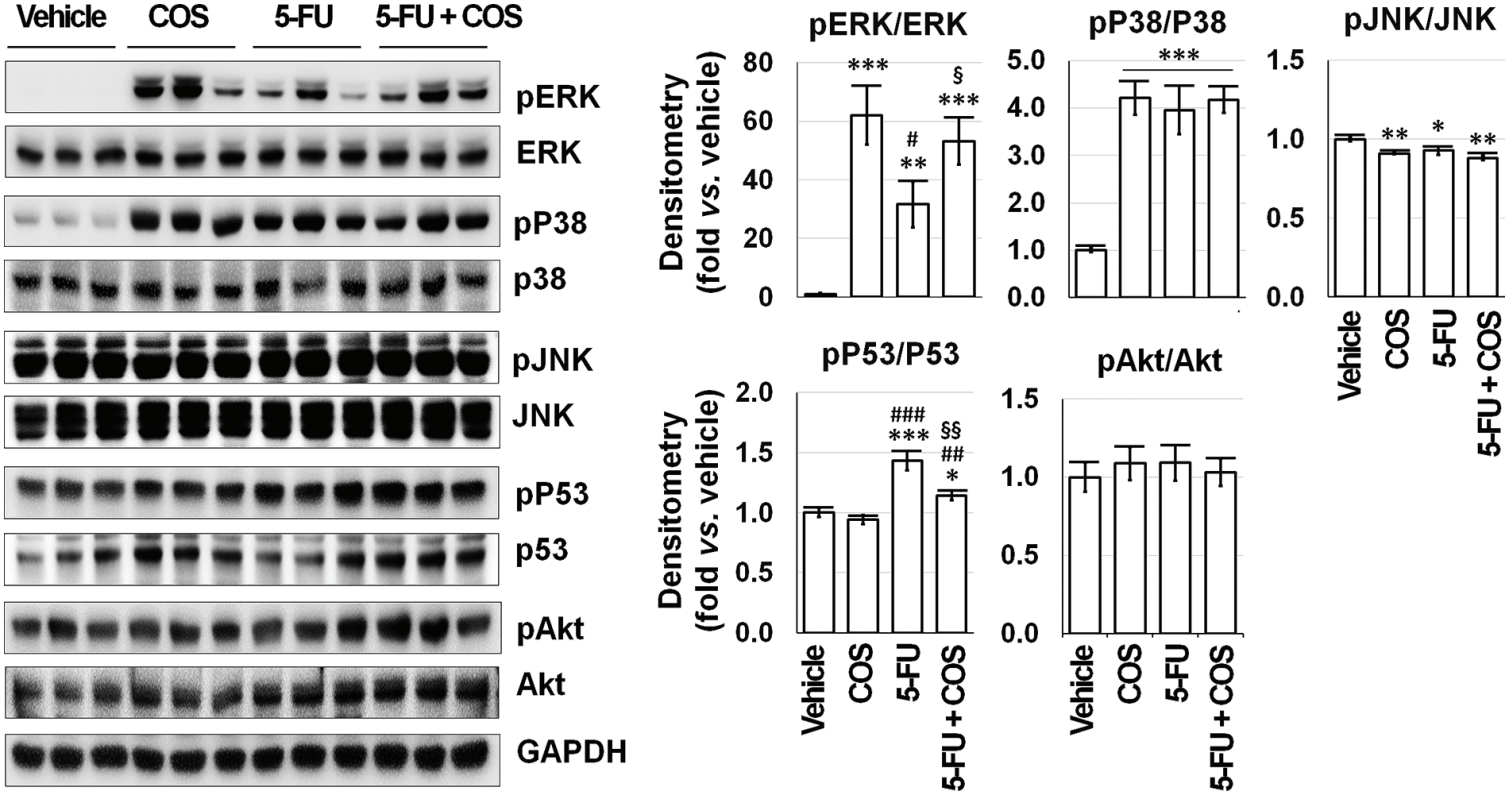
Mitogen-activated protein kinase (MAPK) signaling pathways would be feasible candidates for the mechanism of the anti-cancer effects of chitosan oligosaccharide (COS, 100 μg/mL) in SNU-C5 cells. COS treatment activated ERK with or without 5-fluorouracil (5-FU, 1 μM) among MAPK, p53, and Akt signaling pathways, whereas 5-FU treatment increased p53 phosphorylation. *p < 0.05, **p < 0.01, ***p < 0.001 *vs*. vehicle; ^#^*p* < 0.05, ^##^*p* < 0.01, ^###^*p* < 0.001 *vs*. COS; ^§^*p* < 0.05, ^§§^*p* < 0.01 *vs*. 5-FU.

SNU-C5 cells were treated with vehicle, COS (100 μg/mL), 5-FU (1 μM), and COS plus 5-FU for 24 h, then subjected to Western blotting to identify changes in relevant proteins (Fig. 7). Treatment with COS resulted in significant changes in MAPK, p53, and Akt activation (phosphor/total). Among the MAPK signaling pathways, ERK activation was significantly increased by COS treatment with (*p* = 0.042) or without (*p* < 0.001) 5-FU treatment (62.04 ± 10.06 in COS, 31.64 ± 7.90 in 5-FU, and 53.24 ± 8.03-fold in 5-FU + COS group compared to vehicle; *p* < 0.001). Activation of p38 was significantly increased (4.21 ± 0.35, 3.96 ± 0.51, and 4.18 ± 0.28-fold in each group described above; *p* < 0.001), but the activation of JNK was slightly decreased in all groups. p53 activation was significantly increased by 5-FU treatment, and co-treatment with COS decreased p53 activation (0.94 ± 0.04, 1.43 ± 0.08, and 1.14 ± 0.04-fold; *p* < 0.001).

**Figure 8:**
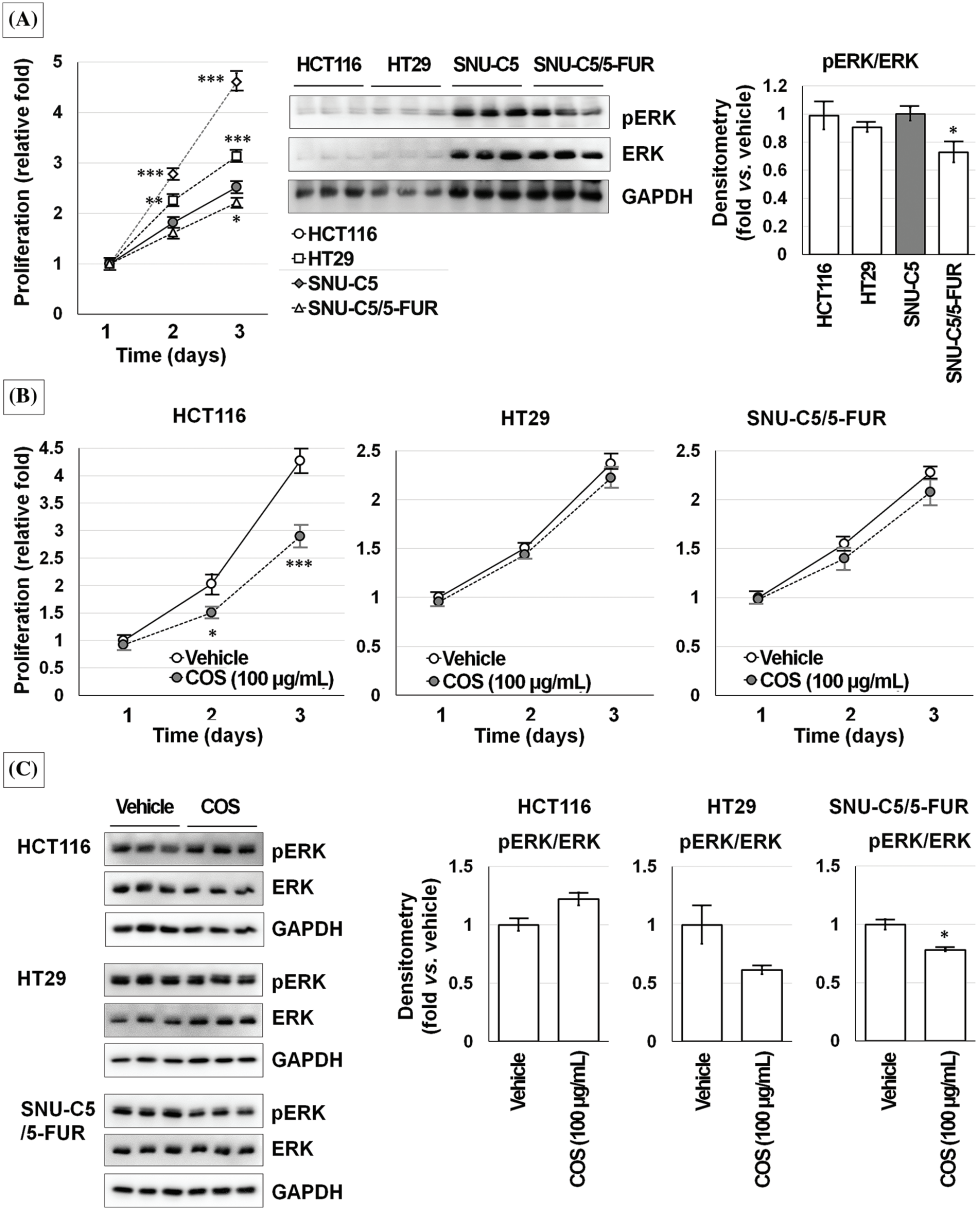
Chitosan oligosaccharide (COS)-induced ERK activation was not observed in other colorectal cancer cells. Although ERK activation was consistent, ERK expression was higher in SNU-C5 cells (A). COS (100 μg/mL) treatment decreased proliferation in HCT116 cells but no significant changes in HT29 and SNU-C5/5-FUR cells (B). COS (100 μg/mL) treatment did not change the ERK activation in HCT116 and HT29 cells but significantly decreased in SNU-C5/5-FUR cells (C). *p < 0.05, **p < 0.01, ***p < 0.001 *vs*. vehicle.

Because the anti-cancer effects of COS were ERK-dependent, we checked ERK activation in other CRC cells (Fig. 8). HCT116 and HT29 cells showed faster proliferation than SNU-C5 and SNU-C5/5-FUR cells. The expressions of ERK and pERK were higher in SNU-C5 and SNU-C5/5-FUR cells than in HCT116 and HT29 cells. COS (100 μg/mL) treatment did not affect the proliferation of HT29 and SNU-C5/5-FUR cells. The relative proliferation of vehicle- and COS (100 μg/mL)-treated HCT116 cells was calculated as 4.27 ± 0.23-fold and 2.90 ± 0.20-fold, respectively, on day 3 (*p* < 0.001). ERK activation was not considerably changed in HCT116 (1.22 ± 0.06-fold; *p* = 0.082) and HT29 (0.61 ± 0.03-fold; *p* = 0.054) cells but significantly decreased in SNU-C5/5-FUR (0.78 ± 0.01-fold; *p* = 0.027) cells.

**Figure 9:**
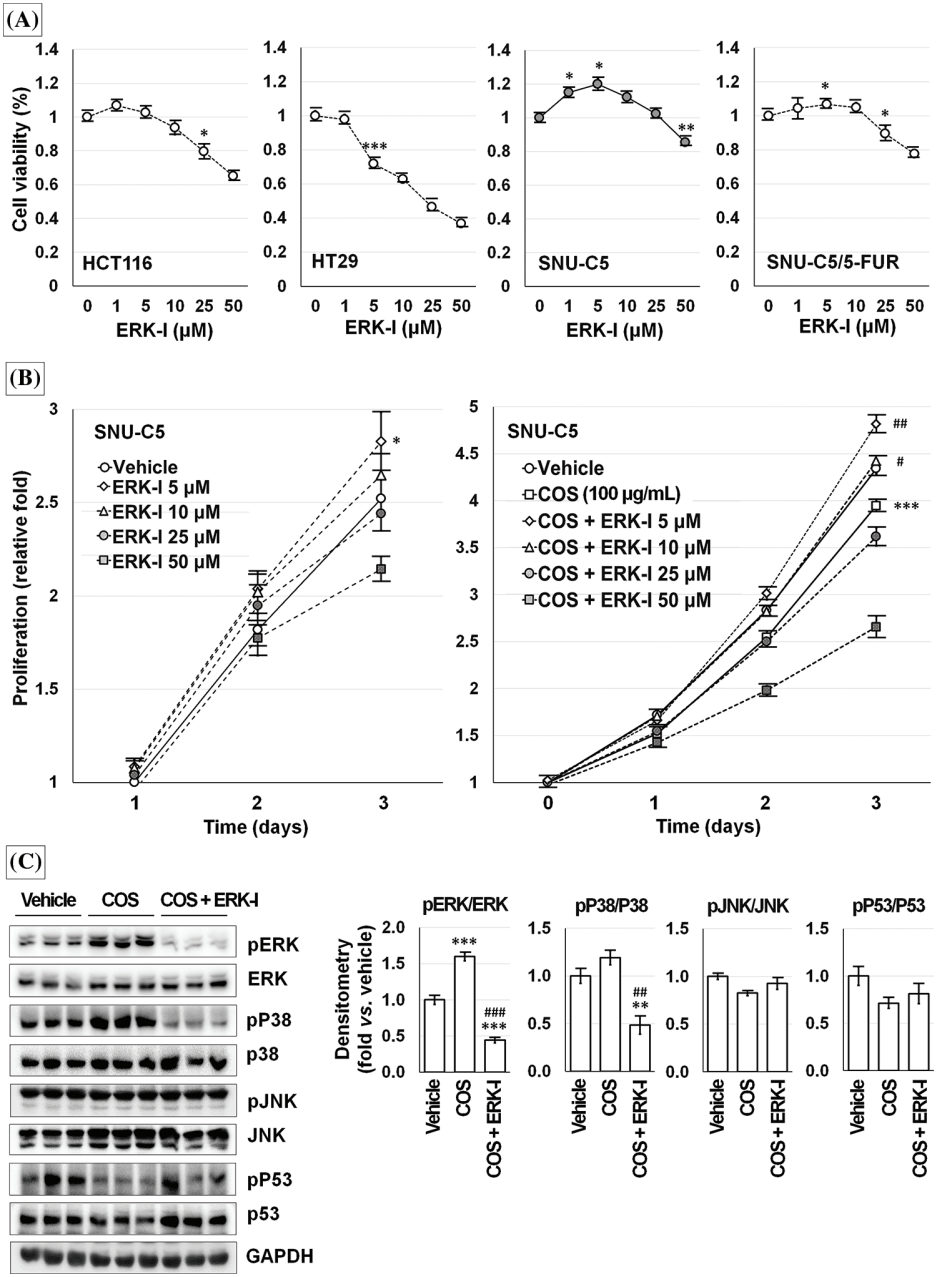
Chitosan oligosaccharide (COS)-induced ERK activation was specific in SNU-C5 cells. ERK inhibitor (ERK-I; PD98059) diminished cell viability in a dose-dependent manner but increased with low-dose in SNU-C5 cells (A). PD98059 significantly increased the relative proliferation at 5 μM. COS (100 μg/mL)-induced anti-proliferative effects were abolished by co-treatment with PD98059 at 5 and 10 μM (B). COS-dependent activation of ERK and p38 was decreased by co-treatment with PD98059 (C). *p < 0.05, ***p < 0.001 *vs*. vehicle; ^#^*p* < 0.05, ^##^*p* < 0.01, ^###^*p* < 0.001 *vs*. COS.

An ERK inhibitor (PD98059) treatment decreased the viability of HCT116 and HT29 cells in a dose-dependent manner. SNU-C5 and SNU-C/5-FUR cells showed slightly increased viability at a low dose and decreased viability at a high dose of PD98059 (Fig. 9A).

SNU-C5 cells proliferated up to 1.82 ± 0.09 and 2.52 ± 0.10-fold at 48 and 72 h as compared with the vehicle-treated condition after 24 h of incubation. Exposure to 5 μM PD98059 increased the relative cell proliferation by 1.08 ± 0.04, 2.04 ± 0.10, and 2.83 ± 0.16-fold at 24 (*p* = 0.043), 48 (*p* = 0.011), and 72 h (*p* = 0.036) compared to vehicle-treated condition. Exposure to 10 μM PD98059 slightly increased the proliferation without significance. The relative proliferation of vehicle-treated SNU-C5 cells was calculated as 4.14 ± 0.19-fold on day 3, which was significantly decreased to 2.34 ± 0.07-fold by COS (100 μg/mL) treatment (*p* < 0.001). Treatment with 5 (3.09 ± 0.17-fold; *p* = 0.007 *vs*. vehicle) and 10 μM (2.82 ± 0.19-fold; *p* = 0.040 *vs*. vehicle) PD98059 and COS enhanced the proliferation. However, cell proliferation was inhibited by 1.46 ± 0.10-fold (*p* = 0.001) at 50 μM (Fig. 9B).

After treating SNU-C5 cells with the vehicle, COS (100 μg/mL), and COS plus PD98059 (10 μM) for 3 days, (Fig. 9C), ERK activation (*p* < 0.001) was significantly increased in cells treated with COS compared to vehicle treatment (1.60 ± 0.06-fold; *p* = 0.001) but decreased with PD98059 co-treatment (0.44 ± 0.04-fold; *p* < 0.001 *vs*. vehicle and *vs*. COS). p38 activation (*p* = 0.003) was decreased by PD98059 treatment (0.48 ± 0.10-fold; *p* = 0.007 *vs*. vehicle, *p* = 0.002 *vs*. COS) but similar in cells treated with COS (1.19 ± 0.07-fold; *p* = 0.077). The activation of JNK and p53 did not change considerably in cells treated with COS and PD98059.

## Discussion

Chitin, chitosan, and COS are known to exhibit biological activities such as antioxidant and anti-cancer effects [8]. In this study, we clearly showed the antiproliferative effects of COS in SNU-C5 cells compared with other CRC cell lines. The anti-proliferative effects of COS were further augmented by co-treatment with 5-FU, which was not observed in other CRC cell lines.

Anti-tumor effects of COS were known to be related to cell cycle arrest [10,15] or apoptosis [13–15]. In this study, water-soluble COS did not affect cell death pathways, including apoptosis and necrosis, whereas 5-FU induced apoptosis. However, COS affected cells cycled pending on the presence of 5-FU. COS treatment without 5-FU significantly increased the fraction of cells in the G0/G1 phase and the fraction of cells in the G2/M phase with 5-FU. Taken together, COS might affect the cell cycle in SNU-C5 cells, which could be related to the anti-proliferative effects.

SNU-C5 cells showed early ERK activation after yeast extract treatment but a significant decrease after 3 days [6]. SNU-C5 cells showed increased ERK activation under basic culture conditions [17], which was also observed in this study as compared to HCT116 and HT29 cells. In this study, among MAPK signaling pathways, ERK activation was observed after COS treatment with or without 5-FU. A high concentration of PD98059 (50 μM in this study) inhibited proliferation, but a low-dose of the ERK inhibitor (up to 10 μM) prevented the anti-proliferative effects of COS in SNU-C5 cells.

The mechanisms of COS involved in the MAPK signaling pathways were reported to differ. COS induced the phosphorylation of MAPKs in RAW 264.7 cells [18,19] and osteoclasts [20]. However, COS repressed MAPK activation in stem cells [21,22] and decreased ERK and JNK but increased the activation of p38 in retinal oxidative damage [23]. In CRCs, COS inhibited ERK activation [24] but did not affect afatinib-induced ERK inhibition [25] in T84 cells. The SNU-C5 cells showed different results, as T84 cells were isolated from CRC lung metastasis. COS induced ERK activation with or without 5-FU and co-treatment with a low-dose of the ERK inhibitor abolished the anti-proliferative effects of COS in SNU-C5 cells.

Previous studies on ERK and the cell cycle in CRCs also showed diverse results. ERK activation resulted in the G0/G1 phase arrest [26] or the G2/M phase arrest [27] on CRC cells. However, ERK deactivation was also related to cell cycle arrest at the G0/G1 phase [28] or the G2/M phase [29]. Likewise, this study clearly showed that ERK activation was specific in COS-induced anti-proliferative effects and that low-dose ERK inhibitors prevented the anti-proliferative effects of COS in SNU-C5 cells. The findings were similar to those in a previous report [30], which showed that ERK activation inhibited proliferation in non-small cell lung cancer.

In our previous report [7], treatment of SNU-C5/5-FUR cells with *Orostachys japonica* induced ERK activation rather than other MAPK signaling pathways and decreased Akt activation, resulting in decreased β-catenin and GSK3β in a xenograft model. Although COS is known to inhibit Akt activation in cancer [31,32], COS did not affect Akt in the SNU-C5 cells in this study. SNU-C5/5-FUR cells showed increased ATP-binding cassette sub-family G member 2 (ABCG2) expression and decreased ERK activation compared with SNU-C5 cells. When ABCG2 expression was inhibited, ERK activation was also inhibited, but proliferation was promoted [17]. Therefore, the relationship between ERK activation and proliferation might be specific to the SNU-C5 cells used in this study.

Multidrug resistance to conventional chemotherapy is still the major challenge in treating CRCs [5]. A combined approach with flavonoids or natural medicine, such as COS and classic anti-cancer chemicals, might be an excellent strategy with fewer toxicity-related problems and enhanced efficacy. MAPK over-activation is well known in several cancers, but ERK activities were lower in CRC tumors than in paired normal tissues [33]. Therefore, the roles of MAPK, especially ERK, in cell growth in CRC are considered both positive and negative. Our results might be reinforced by a previous report [34] that ERK activity mediated anti-proliferative effects depending on the cell type and stimulus. Chitosan and COS have been introduced as drug carriers [32,35,36], and thus, a nano-delivery system with COS would be an alternative approach in cancer research.

This study has the following limitations. First, the enhanced anti-proliferative effects of COS by activating ERK depend on cancer cells, only in SNU-C5 cells and not in HCT116, HT29, and SNU-C5/5-FUR cells. The underlying mechanisms should be further investigated why it is not observed in other cancer cell lines. In addition, a normal colorectal cell line should also be included in the following experimental design to declare the anti-cancer properties of COS. While we suggested ERK activation as a potential mechanism, exploring other signaling pathways or downstream effectors in COS-mediated anti-cancer effects will provide a more nuanced understanding of anti-cancer properties and potential therapeutic targets of COS. Second, the results showed a discrepancy between the effective doses observed *in vitro* and *in vivo* experiments. Therefore, conducting comprehensive dose-ranging studies *in vivo* to establish dose-response relationships and determine the optimal dose of COS for anti-cancer efficacy while minimizing potential adverse effects. This would provide a more robust understanding of the dose-effect relationship and enhance the translational potential of the present findings.

## Conclusion

Taken together, we revealed that COS exerted anti-proliferative effects and enhanced the anti-cancer effects of 5-FU in SNU-C5 cells. The anti-cancer effects of COS with or without 5-FU depend on cell cycle arrest and were related to ERK activation in SNU-C5 cells. However, more specific mechanisms of the anti-cancer effects of COS should be investigated.

## Data Availability

The datasets generated during and/or analyzed during the current study are available from the corresponding author upon reasonable request.

## Abbreviations

COS: Chitosan oligosaccharide
5-FU: 5-fluorouracil
CRC: Colorectal cancer
Akt: Protein kinase B
ERK: Extracellular signal-regulated protein kinase
MAPK: Mitogen activated protein kinase
PD-L1: Programmed cell death-ligand 1
JNK: C-Jun N-terminal kinase
DMSO: Dimethylsulfoxide
ABCG2: ATP-binding cassette sub-family G member 2
